# Co‐Design of a Facilitated Self‐Management Toolkit for People With Parkinson's Disease

**DOI:** 10.1111/hex.70104

**Published:** 2024-11-15

**Authors:** Nathan Davies, Megan Armstrong, Jennifer S. Pigott, Danielle Nimmons, Joy Read, Benjamin Gardner, Bev Maydon, Anette Schrag, Kate Walters

**Affiliations:** ^1^ Centre of Psychiatry and Mental Health Queen Mary University London UK; ^2^ Research Department of Primary Care and Population Health University College London London UK; ^3^ Department of Clinical and Movement Neurosciences University College London London UK; ^4^ School of Psychology University of Surrey Guildford UK; ^5^ Patient and Public Involvement (PPI) London UK

**Keywords:** co‐design, Parkinson's disease, self‐management, toolkit

## Abstract

**Background:**

Parkinson's disease is a complex progressive neurodegenerative disease increasing globally. Self‐management interventions have shown promise in improving the quality of life for people with chronic conditions. This paper aims to describe the development processes and the core components of a facilitated self‐management toolkit to support people with Parkinson's disease to self‐manage their condition.

**Methods:**

An iterative co‐design approach was adopted and included the use of systematic reviews, qualitative methods and theory to develop the Live Well with Parkinson's toolkit. A co‐design group was established consisting of people with Parkinson's, family carers and health and social care experts to produce and refine an online self‐management toolkit to be tested in practice. Five co‐design groups were conducted alongside several phases of toolkit development.

**Results:**

An online self‐management toolkit, called Live Well with Parkinson's, was developed with core aspects such as tailored information to the individual, a well‐being section using an asset‐based approach and problem‐solving skills to create and maintain goals, symptom review and management sections and a tracker of medication, symptoms and activities/actions. A paper‐based alternative version was also created for those who cannot use an online resource. The toolkit is fully manualised and facilitated by six sessions from a ‘supporter’ who is trained in behaviour change techniques. It can be shared with carers and healthcare professionals.

**Conclusion:**

The toolkit is a robust and comprehensive approach to self‐management of Parkinson's disease. It is evidenced based, incorporates theory and was developed with people with Parkinson's and experts in the area. The Live Well with Parkinson's toolkit has the potential to be embedded within routine healthcare, aligning with self‐management policies.

**Patient or Public Contribution:**

Author B.M. is our patient and public involvement (PPI) representative and author on this paper. B.M. supports a team of 15 PPI members who have contributed to the development of the toolkit. This involvement has included attending research team and steering group meetings, attending and facilitating co‐designed workshops, reviewing all the toolkit information before approval and supporting with the user testing.

## 1 Background

Parkinson's disease is a progressive neurodegenerative disease, with the prevalence increasing globally [1] and expected to reach 14.2 million in 2040 [2]. Parkinson's disease is associated with movement and non‐movement symptoms; however, everyone experiences the disease and symptoms differently. Movement symptoms include slowed movement, rigidity, tremor and trouble moving or walking. Non‐movement symptoms include cognitive difficulties, fatigue, dizziness, hallucinations and delusions, pain, sleep disorders and speech and swallowing problems [3, 4]. All these symptoms can impact the quality of life of the person, decrease their independence and increase their dependency on those around them. Many aspects of Parkinson's disease can be managed to relieve symptoms and ultimately improve the quality of life of the individual.

Patient or carer participation in the management of care is increasingly encouraged in health care for long‐term conditions, in particular there has been an increased focus on the use of self‐management to support people living with long‐term conditions [5, 6]. Self‐management has been defined as ‘the care taken by individuals towards their own health and wellbeing: it comprises the actions they take to lead a healthy lifestyle; to meet their social, emotional and psychological needs; to care for their long‐term condition; and to prevent further illness or accidents’ [7]. Self‐management interventions aim to target skills including: problem‐solving, decision making, resource utilisation, forming of patient/health care provider partnership and taking action [6]. Additionally, self‐management has increasingly involved the use of digital interventions including the use of websites, apps for mobile phones and tablets [8]. A recent review identified 36 studies evaluating self‐management interventions for people with Parkinson's disease, with mixed results of approaches and effectiveness of the interventions [9]. There are currently no known effective theory and evidence‐based comprehensive personalised self‐management tools designed for use in routine clinical care (e.g., the NHS in the United Kingdom) to support people with Parkinson's disease.

Co‐design is increasingly used for intervention development in healthcare [10]. Co‐design adopts a partnership‐based approach where end users are integral to and drive the design of the intervention, and work together with the research team [11]. This paper reports the systematic development and components of a tailored self‐management toolkit available online or in paper for people with Parkinson's disease. This builds on a programme of work that includes systematic reviews of evidence for the effectiveness of self‐management interventions [12] and self‐management components as experienced by people with Parkinson's disease and their carers [13]; primary qualitative studies with Parkinson's disease and healthcare professionals (HCPs) [14, 15, 16, 17]; a synthesis of the systematic reviews and qualitative evidence [18] and supplementary reviews that explored advanced care planning [19], alternative and complementary therapies with people with Parkinson's [20] and fidelity and engagement measures of self‐management interventions [21] to inform how they could be incorporated into a self‐management intervention.

## 2 Aim

To co‐design the core components of a facilitated self‐management toolkit to support people with Parkinson's disease living in the community.

## 3 Methods

### 3.1 Design

We used an iterative partnership co‐design method for intervention development [10, 22], following the MRC framework for developing complex interventions [23] and a review of developing complex interventions [11], which provides a comprehensive range of approaches and actions. The actions are mapped across various stages of intervention development and include conception; planning; designing; creating; refining; documenting and planning for future evaluation. These actions were used throughout the work leading up to co‐design workshops and informed the co‐design workshop planning and process. The intervention has been summarised using the TIDieR checklist [24] in our protocol paper for our current Randomised Controlled Trial (RCT) testing the clinical and cost‐effectiveness of this intervention in improving the quality of life in Parkinson's [25].

### 3.2 Theoretical Frameworks Underpinning Development

Alongside the MRC framework and review of complex intervention development approaches and strategies, we used three main underpinning theoretical frameworks to develop the intervention, which were employed alongside the co‐design workshops to develop various aspects of the toolkit. The three theories are:

1. *Self‐management theory*: Self‐management is a problem‐based approach and Corbin and Strauss's model of self‐managing a long‐term condition states that self‐management interventions and programmes must be grounded in the perceptions of patients [26], hence it is important to include them in all seven domains of intervention development as discussed in the review from O'Cathain et al [11]. In Corbin and Strauss's model there are three self‐management tasks to address: medical or behavioural management, physical symptom management and emotional management. A further six self‐management skills have been described, problem‐solving, decision making, resource utilisation, formation of a patient–provider partnership, action planning and self‐tailoring [6]. The premise of self‐management is that teaching participants to actively identify challenges and solve problems associated with their illness themselves with support.
2. *Behaviour change model*: Integral to self‐management is behaviour change. The behaviour change wheel provides a framework for designing behaviour change interventions and offers a theoretical basis to support understanding and the development of self‐management for people with Parkinson's disease [27, 28]. At the centre of this framework is the Capability, Opportunity, Motivation, Behaviour (COM‐B) model, which explains how behaviour is the result of interaction between these former three components. These components are then linked/mapped to intervention components and policy categories to provide the overarching of behavioural change. The behavioural change wheel provides a framework to select appropriate behaviour change techniques to underpin our intervention. We used the behaviour change wheel to select behaviours to target within the three self‐management tasks identified from Corbin and Strauss's self‐management theory [26].
3. *Asset‐based model and person‐centred approach*: By encouraging participants to focus on their assets (i.e., what they enjoy and can do) as opposed to their deficits (i.e., what they cannot do) they may be encouraged to maintain the positive behaviours [29]. Person‐centred care means working in partnership with the participant to develop a plan to address their needs or priorities.
4. *Normalisation process theory* (*NPT*): NPT is a theoretical framework used to understand and explain how new interventions become routinely embedded in health and social care contexts and focuses on four key constructs: coherence, cognitive participation, collective action and reflexive monitoring [30]. Coherence refers to the sense‐making work that individuals and groups undertake to understand the intervention and its benefits. Cognitive participation involves the relational work to engage stakeholders and ensure their buy‐in and commitment. Collective action pertains to the operational work required to enact the intervention within existing systems. Reflexive monitoring encompasses the appraisal work done to assess the intervention's impact and effectiveness over time. NPT provided us with a comprehensive lens to explore the potential barriers and facilitators to the implementation of the toolkit.

### 3.3 Co‐Design Workshop Participants and Recruitment

People with Parkinson's disease and family carers were recruited through neurology outpatient clinics, General Practitioner surgeries and local charity groups (e.g., Parkinson's UK). In addition, members of the study patient and public involvement (PPI) advisory panel were invited to take part. HCPs were purposively sampled to include a wide range of roles across hospital and community settings and levels of experience working with people with Parkinson's disease and were recruited from hospital and community settings across the Greater London region, known contacts of the research team and using snowballing methods.

#### 3.3.1 Inclusion/Exclusion Criteria

People with Parkinson's disease had to have a diagnosis of idiopathic Parkinson's disease and be living in the community. Those with atypical Parkinsonism, living in a care home, lacking capacity to provide informed consent, or had a life expectancy of less than 6 months were excluded. Family carers were excluded if they were less than 18 years of age. HCPs were included if they had experience with working with patients with Parkinson's disease.

### 3.4 Procedure

**Figure 1:**
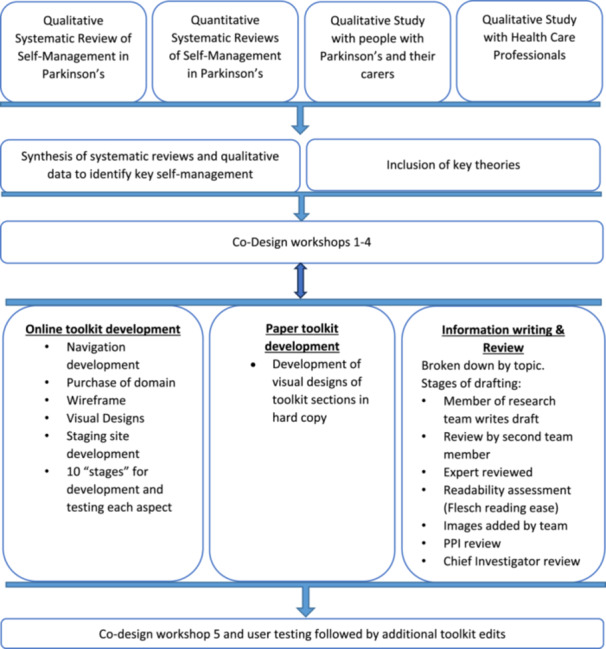
Intervention development process.

The development of the intervention was divided into four main steps which we have aligned with the O'Cathain domains: Step 1—conception and planning: data synthesis; Step 2—designing the toolkit: co‐design workshops; Step 3—development of the toolkit with content and website development teams Step 4—user testing (using a ‘think aloud’ approach) and refinement. Steps 2–3 occurred in parallel to iteratively develop and refine the intervention. An overview of the intervention development process can be seen in Figure 1.

#### 3.4.1 Step 1—Conception and Planning: Data Synthesis and Theoretical Underpinning

The toolkit development was grounded in the above theories and information from qualitative studies with people with Parkinson's disease, family carers and health and care professionals [14, 15, 16], in addition to two systematic reviews of self‐management approaches in Parkinson's disease [12, 13]. Using a matrix approach used in similar co‐design studies [31, 32, 33] and following the methods from Cochrane Qualitative and Implementation Methods Group's guidance on integrating qualitative and quantitative review evidence [34], data from the reviews and qualitative studies was synthesised [18]. The aim was to identify the key required components for our self‐management toolkit for people with Parkinson's disease. Aligning with the actions of the O'Cathain et al. taxonomy this helped us to understand there was a problem in need of a new intervention (i.e., the toolkit); understand the problems or issues to be addressed in the toolkit; identify possible ways of making changes to address the problems; consider real world issues about cost and delivery of the intervention; generate ideas about solutions, components and features of the intervention; and finally, it helped us establish a group for co‐design to help further the development process [11].

#### 3.4.2 Step 2—Designing the Toolkit: Co‐Design Workshops

We convened a co‐design panel consisting of people with Parkinson's disease, family carers/partners, health and care professionals from community and secondary care, volunteer organisations and academic experts. We used a combination of synchronous and asynchronous methods to maximise input from all participants [33, 35]; this included five workshops between July 2019 and March 2020 with a total of 32 HCPs, seven people with Parkinson's, seven family carers, five academics and one patient representative from the voluntary sector (see Table 1). The first four of these workshops were face‐to‐face meetings held at the University, with the final workshop held virtually due to COVID‐19 restrictions; telephone discussions; and written feedback via email or post. The purpose of the co‐design workshops was to understand and clarify the problems or issues to be addressed in the toolkit; make decisions about the specific problems the toolkit aims to address; identify possible ways of making changes to address the problems; consider real world delivery and implementation; generate and refine ideas about solutions, components and features of the toolkit; consider decisions about where to intervene (i.e., target population, scope of toolkit, level at which the toolkit is implemented); make decisions on content format and delivery; design an implementation plan and develop prototypes with the content and website development team [11].

**Table 1:** An overview of the co‐design workshops.

Workshop	Attendees	Aim	Content
1	Total 14 participants: 3 people with Parkinson's, 1 Carer, 4 Parkinson's Disease Nurse Specialists; 2 Geriatricians; 1 Dietician; 1 behaviour change academic, 1 adult social care academic; 1 patient representative from the voluntary sector.	Introduce the research project, provide an overview of work completed to date and identify and prioritise the components of the toolkit.	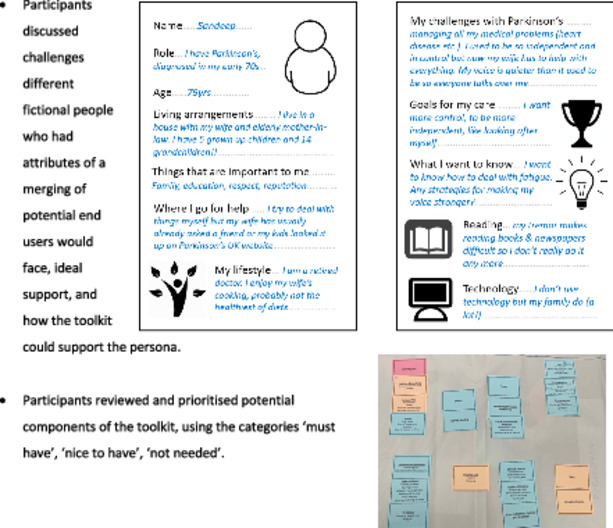
2	Total 20 participants: 4 people with Parkinson's; 1 Carer; 2 General Practitioners (GPs); 2 Physiotherapists; 2 Parkinson's Disease Nurse Specialists; 2 Geriatricians; 2 Occupational Therapist; 1 Dietician; 1 Neuropsychologist; 1 Patient Representative from the voluntary sector; 1 Nursing Academic; and 1 Academic Psychologist.	Define the content, navigation and facilitation of the toolkit.	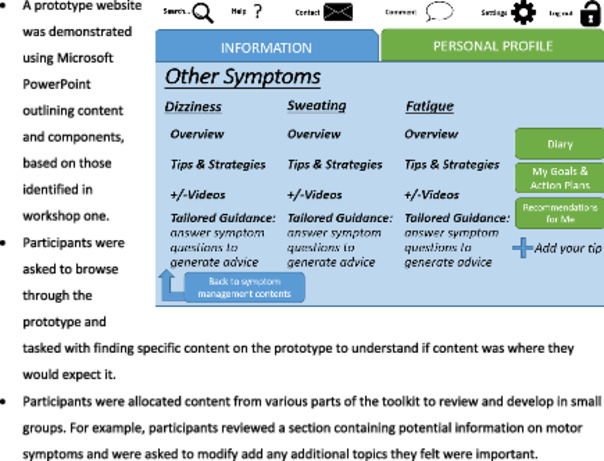
3	Total 7 participants: 3 Carers of people with Parkinson's at advanced stages; 1 Palliative care specialist, 1 GP, 1 geriatrician, 1 psychologist.	Ensure the toolkit addressed the advanced stages of Parkinson's disease.	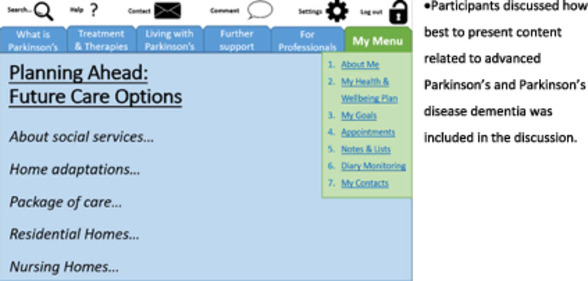
4	Total 23 participants: 5 people with Parkinson's; 1 Carer; 4 Parkinson's Disease Nurse Specialists; 2 Neurologists; 2 Geriatricians; 2 GPs; 2 Physiotherapists; 1 Occupational Therapist; 1 Dietician; 1 Neuropsychologist; 1 patient representative from the voluntary sector; 1 Nursing Academic.	To explore the supporter role, review proposed language and to name the toolkit.	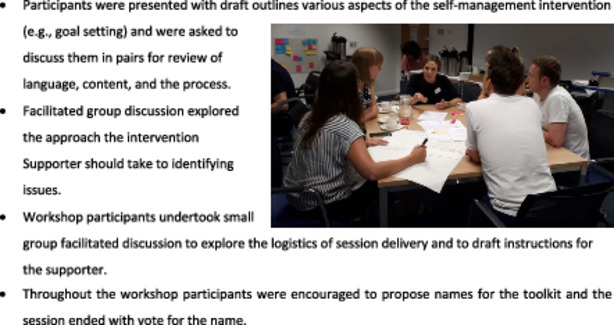
5 (remote)	Total 16 participants: 3 people with Parkinson's; 2 Carers; 2 Neurologists; 2 Community Care (Care Navigator and Frailty Service Case Manager); 1 Occupational Therapist; 1 Foundation Doctor; 2 Psychologist Academics; 1 Nursing Academic.	To demonstrate the full prototype of the website and identify any refinements.	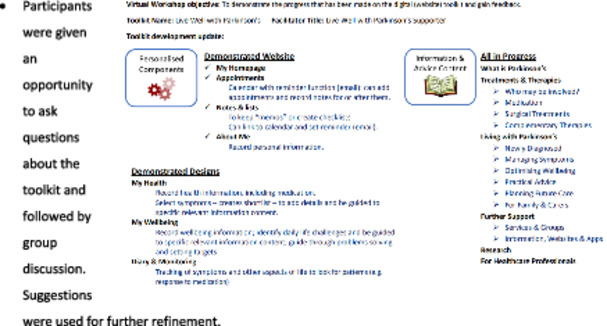

The initial points were delivered by various members of the research team to the larger mixed co‐design group as a PowerPoint presentation with visual aids, open to questions from participants. The concept of each design task was introduced by a member of the research team to the whole group and was performed in pairs or small groups of four to seven, each with at least one workshop facilitator. At the end of each task, a designated member of the smaller groups would feedback to the wider group. Many tasks included writing notes or sorting items, enabling data to be recorded directly by participants. Facilitators also took notes of discussion and observations of reactions.

#### 3.4.3 Step 3—Development of the Toolkit With Content and Website Development Teams

Step 3 worked in parallel with Step 2. In between workshops the research team met to consolidate and summarise the findings of the workshops and worked closely with the content and website team who began prototype development. This was an iterative process with results and prototypes used to inform subsequent workshops. There were several stages of development which included development of the toolkit sections, homepage, menu options, log‐in details and sharing functions (i.e., to enable participants to share the toolkit with a carer or HCP). Each content page was allocated to an expert in the area, and was reviewed for readability level, then by a member of the PPI team, then finally by the chief investigators (K.W., A.S.).

#### 3.4.4 Step 4: Refining and User Testing

Step 4 occurred in parallel with the later stages of Steps 2 and 3. Once the first version of the toolkit was agreed upon by the research team it was tested with end users individually. Eight people with Parkinson's and carers were identified to participate in this who had not contributed to the co‐design workshops. Participants were asked to browse through the different pages and sections of the toolkit and complete set tasks (e.g., completing a form within the toolkit). A ‘think aloud’ approach was used, whereby participants vocalised what they were thinking while performing tasks or solving problems (in this case viewing the prototype website), observed by a member of the research team [36]. User testing was a continuum of co‐design and informed further iterations of the toolkit, feeding back to the website.

## 4 Findings

### 4.1 Live Well With Parkinson's Toolkit

Workshop 1 identified a need for a personalised self‐management toolkit that contained information on Parkinson's, support managing their symptoms and medication and goal setting. Workshop 4 co‐designed the name of this toolkit: ‘Live Well with Parkinson's’. The toolkit needed to be an interactive website and a paper document to ensure maximum reach and to ensure inclusivity. Alongside key self‐management components, the toolkit should contain information on the participant's key contacts, other health conditions, medication, treatments and research involvement. Workshop 3 (on advanced Parkinson's) identified a need for a ‘Planning future Care’ section and specific information for advanced stages (e.g., cognitive impairment and palliative care). The toolkit should allow the participant to have all their information, health care appointments and anything they feel is important all in one place that can be shared with ease. Workshop 5 refined these sections ensuring the toolkit is easy to navigate. Details on the creation and development of the individual sections of the toolkit and the supporter sessions are below.

#### 4.1.1 Information

Workshop 1 identified the need for information on Parkinson's and Workshops 2 and 3 specified the detail of the information pages such as practical and social aspects, emotional wellbeing, non‐motor and motor symptoms, optimising health, palliative care, tips from people with lived experience and signposting. A total of 64 different information sections were identified on what Parkinson's is, symptoms, therapies and treatments, optimising wellbeing and practice advice. The information pages include written information provided by experts, links to other websites and resources, videos summarising the page (as an alternative to reading the page), diverse images and testimonies from people with Parkinson's (identified as important in Workshop 5). Participants can bookmark any of the 64 information pages, which is pulled through to a personalised section.

#### 4.1.2 Symptom Review and Tracking

Workshop 1 identified the need for a symptom review section and was considered important for people at all stages of Parkinson's disease. The toolkit contains a comprehensive list of Parkinson's related symptoms that the participants rate of degree of severity and how much it is bothering them. Each symptom links to the information page related to it allowing them to review self‐management information and when they should seek help. The participant can share this aspect of the toolkit or print the list of the most bothersome symptoms and take this to health care appointments. In keeping with the desire for a flexible approach that came out of the co‐design process, if the participant does not wish to rate all listed symptoms, they do not need to but rather can focus on specific areas should they wish. A tracking section allows participants to track medication and symptoms alongside key activities and identify patterns. This can be useful for those experiencing medication side effects, falls, ‘on’ and ‘off’ periods, sleep or other issues to keep a record of these experiences. The idea is for participants to address a specific question and not to continuously monitor. Different options for presenting this tracker are provided to enable monitoring at an hour‐by‐hour level or day‐by‐day.

#### 4.1.3 Well‐Being

Support making lifestyle changes was identified as important in Workshop 1 and developed in Workshop 4. The toolkit contains a ‘My Well‐being section’ using an asset‐based approach and combines problem‐solving techniques with the COM‐B behaviour change model [27]. The section helps participants identify the health behaviours they would like to maintain or improve, breaks them down into goals and then allows them to review this process. The supporter will use behaviour change techniques such as outcome goal setting, behavioural goal setting, action planning, problem‐solving, reviewing progress (i.e., review outcome goal; discrepancy between current behaviour and goal; review behavioural goal) and provide feedback. The section includes back‐up plans for when there are symptom fluctuations or motivation drops. To be person‐centred, the section does not prescribe goals but encourages the participants to work out what is important to them and what skills (or assets) they already have.

#### 4.1.4 Supporter

Workshop 4 discussed the importance of having a supporter that has a broad understanding of Parkinson's disease, and good communication, IT and coaching skills. The supporter would ideally have a background in healthcare (e.g., psychology, occupational therapy, nursing), social care or third‐sector care (e.g., care navigation, social prescribing). The supporter will be trained in issues related to Parkinson's, using the toolkit and behaviour change techniques such as the ones described in the well‐being section. Should the participant be using the paper toolkit, the supporter identifies key information pages and separates these out into a ‘My information’ folder.

### 4.2 Intervention Components Mapped to Self‐Management Synthesis

Table 2 shows the list of 15 components as identified from the synthesis of data of self‐management in Parkinsons [37] and how they were incorporated into the Live Well with Parkinson's toolkit.

**Table 2:** List of self‐management components for Parkinson's disease and their inclusion in the toolkit.

Component	Live Well with Parkinson's
Access to the right information and advice on Parkinson's	64 Information pages developed by experts and reviewed by the study team and PPI members.
Social and peer support	Information page for peer support
Support with physical exercise	Robust exercise pages developed by physiotherapist and divided into three categories according to ability
Psychological strategies to stay positive	A section on ‘My wellbeing’ to enable the participant to set goals and information pages of how to improve wellbeing.
Increasing motivation (potentially through asset‐based approach)	Used an asset‐based approached throughout, facilitation from the Supporter who is trained in behaviour change approaches and a goal‐setting section.
Access to good quality aids, adaptations and monitoring devices	Information is provided on how to get aids and how to apply for welfare support. No specific aids are included.
Incorporation of participant's personal strategies and skills	The toolkit uses an asset‐based approach and builds on the skills of the participant.
Support with medication management	A tracker enables them to input information about their medication. There are also information pages about medication and treatments.
Support keeping healthy	Information pages dedicated to diet, exercise and well‐being. The supporter can support the participant to create and maintain goals around this.
Ways to self‐monitor	My tracker enables participants to track medication, symptoms and activities and see the interaction between these.
Holistic and person centred (e.g., tailored)	The toolkit comprises several personalised sections and allows for sharing with HCPs to work in partnership with them.
Adaptive to their social context	The supporter empowers the participant to use the toolkit in a way that suits them. Toolkit is available online or in a paper format.
Increase capability and opportunity to self‐manage	The my wellbeing section breaks down goals into manageable steps and addresses whether the participant has the capability and opportunity. Back‐up plans are also created.
Empowering and including carers	The toolkit can be fully shared by carers and carers can attend supporter sessions.
Encouragement of HCPs to engage with self‐management	The toolkit can be shared with HCPs. Leaflets were created for the participant to share with their HCPs that provided information about the toolkit.

## 5 Discussion

### 5.1 Key Findings

We used an iterative co‐design process to develop an online and paper toolkit of self‐management of Parkinson's that incorporated co‐design and has theoretical underpinnings. The toolkit has been developed with the aim of enabling people with Parkinson's to self‐manage and improve their quality of life. The toolkit, for accessibility purposes, is available as a paper document or online and information is provided in writing or through videos. The toolkit can be shared with carers and HCPs to enable decision making to be person‐centred and shared between the person living with Parkinson's and those caring for them. We suggest the toolkit has the potential to be embedded within routine healthcare settings, aligning with current policy (e.g., NHS Long‐Term Plan) that emphasises self‐management [38].

One of the key aspects of the toolkit, developed in consultation with co‐design workshops and based in theory, is access to accurate, tailored information about their condition. Parkinson's disease is a complex and varied condition, and each individual's experience with the disease can differ substantially. Providing tailored information that is specific to an individual's needs and symptoms can help them better understand their condition, the potential progression of the disease and the various treatment options available. It can also help individuals with Parkinson's be more proactive in managing their symptoms, such as by making lifestyle changes, taking medications as prescribed and engaging in appropriate exercise and wellbeing activities. Research has consistently shown educating people on their conditions is beneficial to helping people self‐manage their chronic diseases [39].

Another key aspect of self‐management is maintaining or improving well‐being. The importance of this aspect has threaded through all of the evidence from the perspectives of people with Parkinson's [13, 16]. Setting realistic and asset‐based goals is crucial for maintaining a sense of control and independence over their lives. Asset‐based approaches empower people to engage in healthy behaviours and due to this empowerment and person‐centred approach, it is an inclusive approach [40]. SMART goals help individuals avoid frustration and disappointment that can occur when setting overly ambitious goals that are difficult to achieve. The effectiveness of using of behaviour change techniques to support participants in their goal development and maintenance has been widely documented [28] and employed in the toolkit as well as in the supporter sessions.

The progressive and fluctuating nature of Parkinson's is one of the biggest hurdles to self‐management in Parkinson's, which can be addressed through symptom and medication tracking [16]. The Live Well with Parkinson's toolkit incorporates tracking of these, as well allowing participants to explore the interplay between medication, symptoms and activities. Tracking can also help individuals communicate more effectively with their healthcare providers, by providing them with accurate and detailed information about their symptoms and medication use. This information can help healthcare providers make more informed decisions about treatment and medication changes.

### 5.2 Strengths and Limitations

A strength of this study is its comprehensiveness in terms of several rounds of iterations with the co‐design group and user testing (specific elements in the initial phases and prototype toolkits in the digital development phase). Feedback from people with Parkinson's, health and social care practitioners and experts in the area was embedded at each stage. For example, each information page was written and reviewed at least five times, including at least three clinicians and someone with lived experience. Additionally, one workshop was specifically focused on the advanced stages of Parkinson's, ensuring the final version of the toolkit was suitable for all disease stages. This robust procedure has enhanced the trustworthiness, design and usability of the toolkit. While there were five workshops that incorporated a range of people with Parkinson's disease (e.g., at various stages), there was not a specific focus on underserved groups and the toolkit needs testing specifically with those groups to ensure inclusivity and appropriateness.

### 5.3 Future Research

The toolkit has been tested in a feasibility study and was confirmed to be an acceptable and valued tool for people with Parkinson's to self‐manage. The Toolkit is now being tested in a fully‐power RCT in the United Kingdom [25].

## 6 Conclusion

The Live Well with Parkinson's toolkit is a novel, digital and paper alternative, intervention for people at all stages of Parkinson's that has been robustly co‐designed. It is hoped this toolkit can be adopted into healthcare settings following testing for effectiveness and will support people with Parkinson's to live independently with a better quality of life.

## Author Contributions

**Nathan Davies:** conceptualisation, investigation, funding acquisition, writing–original draft, writing–review and editing, supervision, formal analysis, data curation. **Megan Armstrong:** supervision, project administration, writing–review and editing, writing–original draft, investigation, methodology, formal analysis. **Jennifer S. Pigott:** writing–review and editing, methodology, formal analysis, data curation. **Danielle Nimmons:** writing–review and editing, methodology, formal analysis, data curation. **Joy Read:** writing–review and editing, methodology, project administration, formal analysis, data curation. **Benjamin Gardner:** conceptualisation, investigation, funding acquisition, writing–review and editing, supervision, formal analysis, data curation. **Bev Maydon:** conceptualisation, investigation, funding acquisition, writing–review and editing, methodology, project administration, formal analysis, data curation. **Anette Schrag and Kate Walters:** conceptualisation, investigation, funding acquisition, writing–review and editing, methodology, project administration, supervision, formal analysis, data curation.

## Ethics Statement

All procedures were approved by the Cardiff University, School of Psychology Ethics Committee [EC.18.03.13.5263].

## Conflicts of Interest

The authors declare no conflicts of interest.

## Data Availability

Further data are available by reasonable request to the corresponding author.
